# Travelers’ knowledge, attitudes, and behavior related to infectious diseases in Italy

**DOI:** 10.1371/journal.pone.0215252

**Published:** 2019-04-12

**Authors:** Abdoulkader Ali Adou, Francesco Napolitano, Alessandra Vastola, Italo Francesco Angelillo

**Affiliations:** Department of Experimental Medicine, University of Campania “Luigi Vanvitelli”, Via Luciano Armanni, Naples, Italy; Amala Institute of Medical Sciences, INDIA

## Abstract

The objectives of this investigation were to examine the travelers’ knowledge, attitudes, and behavior about travel-related diseases and to evaluate the factors that influence their knowledge, attitudes, and behavior. A cross-sectional study was performed between May and September 2018 among a random sample of individuals attending randomly selected travel agencies in the geographical areas of Caserta and Naples, Italy. One fourth of participants (25.6%) had a poor level of knowledge, 50.2% a moderate, and only 24.2% a good level about the most common infectious diseases in the destination country. Those who had received information from physicians about the most common infectious diseases in the destination country and who do not need additional information were significantly more likely to have a good level of knowledge. A large majority (91%) showed no concern about the risk of getting an infectious disease during the travel. Almost half of the respondents had received information concerning the most common infectious diseases in the destination country and the related prevention measures. This information was more likely acquired by those graduated, those who know the foods that can cause the infectious diseases, and those who self-perceived a well health status, and less likely by those who had a poor level of knowledge about the most common infectious diseases in the destination country and who were going to Asia and South America. Education and communication activities regarding all aspects of travel-related diseases are needed to increase the knowledge and the access to preventive measures.

## Introduction

The annual number of international travelers has steadily increased over the last decade all around the world [1] and the acquisition of travel-associated infectious and non-infectious diseases is one of the major public health consequences for them [2,3]. In particular, travel to tropical and sub-tropical geographic areas cause an increased probability of exposure to endemic infectious diseases [4–6]. Moreover, it is well known that the diffusion of travel-related infectious diseases is favored by a higher likelihood of visiting rural and remote areas, activities during travel, greater consumption of high-risk food and drink, and lower likelihood of seeking medical consultation prior to their journey or follow recommended vaccinations [7–10]. For these reasons, it is increasingly important that travelers of high-risk international trips know the impact of infectious and non-infectious diseases in these countries and have a real awareness of the risks of acquiring a travel-related illness, as many of these diseases can be prevented through vaccinations, antimicrobial drugs, and advices from health-care professionals. The World Health Organization (WHO) recommends that travelers, before departure, should be advised about the risk of disease in the country or countries they plan to visit and the steps to be taken to prevent the associated illness [2]. In Italy, travel health counseling are usually delivered in general medical practices.

In this context, understanding the travelers’ knowledge, attitudes, and behavior could provide interesting information to policy makers in order to plan educational interventions on this population to effectively prevent travel-related diseases. The analysis of the literature showed that previous epidemiological research have been conducted in different communities to assess the knowledge and behavior of individuals on the risk of diseases acquired during travel [11–16], but, to the best of our knowledge, currently information on this topic in Italy is still lacking for the absence of published data. Therefore, to address this knowledge gap in the literature, the primary objective of the present investigation conducted in Italy was to examine the travelers’ knowledge, attitudes, and behavior about travel-related diseases and the secondary objective was to evaluate the factors that influence their knowledge, attitudes, and behavior.

## Materials and methods

### Study setting and sample

This cross-sectional study was performed between May and September 2018 in the geographical areas of Caserta and Naples, Italy. A two-stage cluster sampling method has been used to select the participants. In the first stage, five travel agencies were randomly selected from a list of agencies in the geographical areas. At the second stage, a sample of individuals from each chosen agency was randomly selected. Subjects were included in the study if they met the eligibility criteria: (1) were at least 18 years of age; (2) were travelling to Africa, South America, and Asia; and (3) were not resident in the destination country.

The sample size was determined by assuming 30% of expected proportion of having a good level of knowledge, 95% confidence interval, 5% margin of error, a design effect of one was incorporated due to the cluster sampling method, and a non-response rate of 20%. Hence, the total sample size was estimated at 403 participants

### Procedure

Prior to the study’s inception, the managers of the selected travel agencies received a letter to request their collaboration and they were informed about the study’s aims and methodology of the data collection. After the consent obtained from all agencies, an information letter was provided by the research team to the randomly selected participants attending the agencies highlighting the institution availing the study, the importance of their collaboration, the purposes of the study, the data collection methods, the voluntary nature of participation, the confidentiality of all information provided, and the informed consent form to read and sign if they were willing to participate in the study. Participants’ written informed consent was obtained at the beginning of the survey. After obtaining the consent, the questionnaire was administered via face-to-face interviews in the agencies by the personnel of the travel agencies who were trained in data collecting techniques in order to reduce biases. The anonymity and confidentiality of the information were maintained by excluding personal identifiers from the questionnaire, and all the data collected were processed and analyzed anonymously. The interviewees did not receive any financial or other compensation for participation in the study.

### Questionnaire

A pilot study of the questionnaire was carried out with 25 travelers to ensure the clarity and the applicability of the questionnaire, to identify the possible problems that may hinder data collection, and to perform any required changes. The pilot sample was excluded from the final study sample size. The internal reliability was assessed using Cronbach’s α [17]. The final version of the questionnaire (S1 File) consisted of 25 items related to five main categories: (i) socio-demographic (gender, age, educational level, marital status, number of children, employment status, self-rated health status) and travel (destination, reasons, length of stay, number of co-travelers, previous travels) characteristics; (ii) knowledge about the most common infectious diseases in the destination country, causes, and preventive measures. The knowledge has been evaluated using the information reports provided by World Health Organization [18]. All response options of the eleven questions used to assess this knowledge included “yes”, “no” and “do not know”. Knowledge scores for these questions had a value of ‘1’ for each correct response and a value of ‘0’ for each incorrect or ‘do not know’ response. So the cumulative score would range from zero to 11 points for a given participant. A respondent who achieved a composite score greater than or equal to 8 was categorized as a good level of knowledge, 4–7 score as moderate knowledge, and 0–3 as poor knowledge; (iii) attitude towards the travel-related infectious diseases and preventive measures (concern of contracting infectious diseases and usefulness of the preventive measures). Responses to questions related to attitude were graded on a 10-point Likert type scale, anchored by 1 (minimum score) to 10 (maximum score); (iv) behaviors regarding the travel-related infectious diseases (preventive measures, and willingness to undergo preventive measures). Response options included “yes” and “no”, and for each response a choice from a list of reasons; (v) sources of information about travel-related infectious diseases. Response options included “yes” and “no”, and for each response a choice from a list of options.

The Ethical Committee of the Teaching Hospital of the University of Campania “Luigi Vanvitelli” approved the study (n.267, April 6, 2018).

### Statistical analysis

The statistical analysis was performed in several steps (S2 File). First, a descriptive analysis was conducted to summarize the principal characteristics of the participants. Second, a univariate analysis, using chi-square for categorical variables and Student’s t-tests for continuous variables normally distributed, to assess the association between the outcomes of interest and the independents variables and the factors with a *p*-value less or equal than 0.25 were introduced in the multivariate ordered and logistic regression models. Third, multivariate stepwise logistic and ordered logistic regression analyses were performed to investigate the effect of each independent variable on the following outcomes of interest: travelers’ knowledge regarding the infectious diseases in the destination country (Model 1), travelers who considered dangerous to contract an infectious disease while traveling (Model 2), travelers who have received information about the infectious diseases in the destination country and the related prevention measures (Model 3), and travelers’ need of more information about the travel-related infectious diseases (Model 4). The following predictor variables were included in all Models: age (continuous), gender (male = 0; female = 1), marital status (unmarried = 0; married = 1), educational level (high school or lower = 0; baccalaureate degree or higher = 1), at least one parent who is a health care professional (no = 0; yes = 1), self-reported health status (continuous), and previous travels in the destination country (no = 0; yes = 1). The variables source of information about the travel-related infectious diseases (none/travel agency/internet/friends/relatives = 0; physicians = 1), and need of more information about travel-related infectious diseases were included in Model 1; the variables destination country (North Africa = 1; East Africa = 2; Asia = 3; South America = 4), level of knowledge regarding the infectious diseases in the destination country (poor = 1; moderate = 2; good = 3), and correct knowledge about the foods that can cause the infectious diseases in the destination country (no = 0; yes = 1) were included in the Models 2, 3, and 4; the variables considering dangerous contracting an infectious disease while traveling (no = 0; yes = 1) and considering useful the preventive measures for the travel-related infectious diseases (no = 0; yes = 1) were included in Models 3 and 4; the variable being informed about infectious diseases in the destination country and about the related prevention measures (no = 0; yes = 1) was included in Model 4. The significance level was set at 0.2 for entering and at 0.4 for removing the variables in the stepwise logistic regression models. Odds ratios (OR) and their 95% confidence interval (CI) were estimated from the logistic regression models. All statistical tests were two-tailed, and at a *p*-value less than or equal to 0.05 was selected as the cutoff for statistical significance. The Stata statistical software version 15 was used to analyze the data [19].

## Results

### Participants’ characteristics

The results of the pilot study showed a good internal consistency with a Cronbach’s α of 0.75. In total, data of 422 participants out of the 510 selected were analyzed with a response rate of 82.7%. Socio-demographic and general characteristics of the respondents are shown in Table 1. More than half of the sample was male, the average age was 31.4 years, almost all were of Italian nationality, one in four were married (23.8%), 20.5% had at least one child, one third had a graduate degree, almost all were employed, more than two thirds were going to Asia and Africa, 9.3% were business travelers, and only 6% traveled alone.

**Table 1:** Socio-demographic characteristics of the study population.

	N	%
Gender		
Male	220	52.3
Female	201	47.7
Age, mean±SD (range), *years*	31.4±6.6 (15–74)	
Marital status		
Single	240	57
Married	100	23.8
Cohabitant	72	17.1
Separated/Divorced/Widowed	9	2.1
Number of children		
0	329	79.5
≥1	85	21.7
Self-reported health status, mean±SD (range)	8.9±1.2 (1–10)	
Educational level		
Illiterate	1	0.3
Primary school	1	0.3
Middle school	31	7.4
High school	260	62.3
Baccalaureate degree or higher	124	29.7
Employment status		
Employed	409	96.9
Unemployed	13	3.1
Destination country		
North Africa	150	35.6
Asia	137	32.5
South America	106	25
East Africa	29	6.9
Reason of the travel		
Holiday	380	90.7
Business	39	9.3

Number for each item may not add up to total number of study population due to missing value

### Knowledge about travel-related infectious diseases

The questions on the knowledge about the most common infectious diseases in the destination country of their travel, showed that only 16 participants (3.8%) gave all correct answers, and 25.6% had a poor level of knowledge with a mean score of 1.2(±1.2), 50.2% a moderate with a mean score of 5.7(±1.1), and only 24.2% a good level with a mean score of 9.1(±1.1). Moreover, more than half (57.8%) had a correct knowledge about the foods that can cause the infectious diseases in the destination country and only 11% knew the preventive measures of the infectious diseases that were recommended for their destination.

Table 2 presents the factors predictive towards the different outcomes of interest by the use of multivariate ordered logistic and logistic regression analysis. Considering the good level of knowledge about the most common infectious diseases in the destination country as explanatory variable, after adjustment for other covariates, the results of the ordered logistic regression model identified that respondents who had received information from physicians about the most common infectious diseases in the destination country (OR = 2.21: 95% CI 1.39–3.52) and those who do not need additional information (OR = 0.16; 95% CI 0.09–0.29) were significantly more likely to have this knowledge (Model 1).

**Table 2:** Multivariable ordered and logistic regression models indicating associations between independent variables and the outcomes of interest.

Variable	OR	SE	95% CI	*p* value
**Model 1. Travelers’ knowledge regarding the infectious diseases in the destination country**				
Log likelihood = -385-15, χ^2^ = 82.04 (6 df), *p* = 0.0001				
No need of more information about travel-related infectious diseases	0.16	0.05	0.09–0.29	<0.001
Having received information from physicians	2.21	0.52	1.39–3.52	0.001
Previous travels in the destination country	1.69	0.46	0.98–2.89	0.057
Female	1.37	0.26	0.94–2.01	0.104
Self-reported health status	0.96	0.09	0.79–1.16	0.692
At least one parent who is a health care professional	1.04	0.36	0.53–2.04	0.902
**Model 2. Travelers who considered dangerous to contract an infectious disease while traveling**				
Log likelihood = -101.7, χ^2^ = 44.37 (5 df), *p*<0.0001				
Self-reported health status	0.57	0.08	0.43–0.76	<0.001
Level of knowledge regarding the infectious diseases in the destination country				
Good	1*			
Poor	3.55	1.36	1.68–7.51	0.001
Correct knowledge about the foods that can cause the infectious diseases in the destination country	2.87	1.23	1.24–6.64	0.014
Baccalaureate degree or higher	0.65	0.31	0.25–1.66	0.369
Married	1.42	0.56	0.66–3.07	0.372
**Model 3. Travelers who have received information about the infectious diseases in the destination country and the related prevention measures**				
Log likelihood = -204.07, χ^2^ = 148.65 (8 df), *p*<0.0001				
Self-reported health status	2.13	0.27	1.66–2.72	<0.001
Baccalaureate degree or higher	3.29	1.03	1.78–6.06	<0.001
Level of knowledge regarding the infectious diseases in the destination country				
Good	1*			
Poor	0.27	0.08	0.15–0.49	<0.001
Correct knowledge about the foods that can cause the infectious diseases in the destination country	2.18	0.59	1.28–3.71	0.004
Destination country				
North Africa	1*			
Asia	0.42	0.14	0.23–0.8	0.008
South America	0.43	0.15	0.22–0.85	0.015
Previous travels in the destination country	1.61	0.55	0.82–3.13	0.165
Married	1.41	0.39	0.82–2.42	0.214
**Model 4. Travelers’ need of more information about the travel-related infectious diseases**				
Log likelihood = -137.27, χ^2^ = 99.11 (7 df), *p*<0.0001				
Level of knowledge regarding the infectious diseases in the destination country				
Good	1*			
Poor	11.9	6.86	3.89–36.8	<0.001
Moderate	2.81	1.63	0.89–8.77	0.076
Travelers who considered dangerous to contract an infectious disease while traveling	4.08	1.75	1.76–9.48	0.001
Travelers who have received information about the infectious diseases in the destination country and the related prevention measures	0.26	0.1	0.12–0.57	0.001
Self-reported health status	0.83	0.1	0.65–1.05	0.125
Married	1.51	0.49	0.79–2.87	0.204
Baccalaureate degree or higher	0.67	0.26	0.31–1.43	0.3

*Reference category

### Attitudes about travel-related infectious diseases

Regarding the attitudes, it was found respectively that 91% showed no concern about the risk of getting an infectious disease during the travel, with an overall mean score of 2.5(±1.8), and only 12.9% considered useful the prevention measures for infectious diseases before the departure, with a mean score of 2.6(±2.1), out of a maximum score of 10. Using the concern about the risk of getting an infectious disease during their travel as outcome, multivariate logistic regression analysis was conducted to determine which independent variables were significantly associated. The results showed that the respondents with a good level of knowledge about the foods that can cause the infectious diseases (OR = 2.87; 95% CI 1.24–6.64), those with a poor level of knowledge about the most common infectious diseases in the destination country (OR = 3.55; 95% CI 1.68–7.51), and those who self-perceived a worse health status (OR = 0.57; 95% CI 0.43–0.76) were more likely to have this concern (Model 2 in Table 2).

### Practices about travel-related infectious diseases

Almost all participants (93.4%) said that they did not have received any recommendation regarding the measures for preventing the risk of getting an infectious disease in the country where they were going and 93.3% did not practice any preventive measure recommended for their destination country. The primary reasons for not performing any preventive measures were that the respondents believed that such measures were not necessary (84.1%) and that they did not feel to be at risk (14.5%). Among participants who had practiced preventive measures, 45.8% reported that they had received pre-travel vaccinations recommended for the destination country, 41.7% had taken medications to prevent malaria, and 12.5% traveled with a first aid kit.

### Sources of information

Almost half of the respondents said that they had received information concerning the most common infectious diseases in the destination country (49.3%) and travel agencies were the preferred method of updating their knowledge (41.7%), followed by physicians (35.6%), and internet (9.1%). Moreover, 49% had acquired information on the prevention of the infectious diseases. The logistic regression analysis showed that those with a graduate degree (OR = 3.29; 95% CI 1.78–6.06), those with a correct knowledge about the foods that can cause the infectious diseases (OR = 2.18; 95% CI 1.28–3.71), and those who self-perceived a well health status (OR = 2.13; 95% CI 1.66–2.72) were significantly more likely to acquire information about the most common infectious diseases and the related preventive measures, whereas those who had a poor level of knowledge about the most common infectious diseases in the destination country (OR = 0.27; 95% CI = 0.15–0.49), and those who were going to Asia (OR = 0.42; 95% CI = 0.23–0.8) and South America (OR = 0.43; 95% CI = 0.22–0.85) compared to those who were going to North Africa were less likely to acquire such information (Model 3 in Table 2).

Only 17.3% reported that they felt the need to receive additional information on infectious diseases in the destination country. The results of the multivariate logistic regression showed that the factors independently associated with the need to receive such information included a poor level of knowledge about the most common infectious diseases in the destination country (OR = 11.9; 95% CI = 3.89–36.8) and considering dangerous contracting an infectious disease while traveling (OR = 4.08; 95% CI = 1.76–9.48). Moreover, participants who had received information about the infectious diseases in the destination country and the related prevention measures (OR = 0.26; 95% CI = 0.12–0.57) were less likely to feel the need to receive additional information (Model 4 in Table 2).

## Discussion

To our knowledge, ours is the first investigation that aimed at describing the knowledge, attitudes, and behavior about travel-related diseases and the potential role played by several factors among travelers in Italy and the findings could offer an insight on the strategies required to effectively address this important public health theme.

Generally speaking, the need to improve the level of knowledge is clearly stated in this study that indicates that the proportion of surveyed participants who were knowledgeable about the most common infectious diseases in the destination country of their travel was found to be strikingly low with only 24.2% having a high level. This result is quite disturbing and it is explicitly unsatisfactory compared with surveys previously published from other countries [11,12,20]. These differences may be partly attributed to the characteristics of the population samples, methods of collecting the data, and instruments used. Of particular concern is the fact that the vast majority of travelers did not have received any recommendation regarding the measures for preventing the risk of getting an infectious disease in the destination country and consequently did not practice any preventive measure before their travel. The results of this study did not reinforce the findings of several previous studies conducted in other countries that highlight the very frequent health advice before travelling use from a doctor [10,14,15,21].

It is important to note that a large majority of the participants showed no concern about the risk of getting an infectious disease during their travel. This result is in line with previous investigations conducted in other countries and on different populations that have underlined the low perception of travelers about the health risks [22–25].

Results from this study indicated that a substantial proportion of respondents did not receive recommendation regarding the measures for preventing the risk of getting an infectious disease in the destination country and, therefore, the question at hand is how to include these insights into practice. Moreover, the vast majority of the sample did not practice any preventive measure before their travel and the predominant reasons were that they believed that the measures were not necessary and that they did not feel to be at risk. These reasons are consistent with those observed in previous investigations [26,27]. In this study the most common preventive measures performed by participants were pre-travel vaccinations, medications to prevent malaria, and travelling with a first aid kit. Similar findings were reported in already cited studies [11,12]. In general, this misconception and the fact that travelers do not usually undergo health checkups until they experience health problems may help explain the poor access. Moreover, the absence of systematic and active promotion of a preventive program may contribute to this low utilization and therefore it is critical to raise awareness regarding the importance of regular screening in this population.

The multivariate logistic regression analysis revealed several socio-demographic characteristics of participants associated with the different outcomes of interest. It has been found that those graduated were significantly more likely to have acquired information about the most common infectious diseases and the related preventive measures. Moreover, of note, as expected, the perception of health status was associated with attitudes since those who perceived a lower level of health were more likely to believe that they were at risk of getting an infectious disease during their travel. In this study, having a poor level of knowledge about the most common infectious diseases in the destination country was associated with the perception of the risk of getting the disease during the travel.

Understanding the sources of information could assist public health policy makers to design interventions to improve mainly the level of knowledge and an appropriate behavior. The travel agencies were indicated as primary and most trusted source of information concerning the most common diseases in the destination country and this may partially due because they can be accessed most easily during the organization of a travel. However, this source cannot be considered as a replacement for information and advice from physicians. Indeed, physicians seem to be ideal ground actors for the population’s education. That said, despite worldwide acknowledgment that physicians are key, it is important to draw attention to the discouraging aspect that in the present study, physicians were recognized by one-third of participants as a source of information of the most common infectious diseases in the destination country. Therefore, given that the second most-common source was physicians, interventions should be targeted toward improving access to healthcare in the future. Moreover, an important finding of the multivariate analysis of this study was that physicians can influence the knowledge in the respondents since physician’s direct advice was significantly associated with the good level of knowledge and those who have not ever discussed with them were less knowledgeable than counterparts. This might be explained by the fact that public receive incomplete information depending on the source from which they seek it. Previous studies conducted by some of us among different groups of the general population in other contexts demonstrated the fundamental role that healthcare workers play in communication strategies and educational campaigns in order to improve knowledge, attitudes, and practices [28–32]. Just as previous studies have shown, this study also confirms that their recommendations are significantly associated with the likelihood of improvement in the level of knowledge and in the appropriate behavior [30–34]. Furthermore, this study reinforces once again the importance for new comprehensive approaches or public health policies and programs and encouraging healthcare professionals to act as an information source in order to improve the quality of their relationship with the population and to consequently increase the level of knowledge. Most interestingly, it is noteworthy that only half of the participants had acquired information concerning the most common infectious diseases in the destination country and this result is lower than those showed by studies previously conducted in Europe among travelers [14,15,22,35]. Moreover, only 17.3% expressed a strong desire for obtaining additional information.

As with all studies, it is worth mentioning here that a few limitations of the present study bear emphasizing in terms of the research design and data collection method. First, due to the cross-sectional methodology, the directionality of the association or the causal relationship between the knowledge, attitude, and practice of travel-related diseases and the characteristics could not be explored in depth; however, the findings provide a basis for acquiring and testing a causal hypothesis. Second, this study was carried out in one geographic area of Italy and so, generalization of the results should be made with caution. Third, there is the possibility of recall bias, as we were asking question related to the past, even if not so distant. It is quite possible that respondents were not able to accurately remember the details that they were asked about. Fourth, surveys often raise concerns about social desirability bias, though we tried to limit this by collecting the data with the personnel of the travel agencies. Fifth, history of practice of preventive measures was based on participants’ self-reported information and a likelihood of recall bias cannot be ruled out. In spite of these limitations, as the first survey of its kind from Italy, the current study provided useful information of the knowledge, attitudes, and behavior toward travel-related diseases and factors of influence among travelers.

## Conclusions

The results of the current study showed that travelers had a low level of knowledge related to infectious diseases and rarely practiced preventive measure before their travel. These findings underlined the importance that healthcare workers should implement clear education and communication activities regarding all aspects of travel-related diseases to increase the knowledge and the access to preventive measures.

## Supporting information

S1 File Questionnaire. (DOCX)

S2 File Data file. (XLSX)
