# Supplementary material for: Travelers’ knowledge, attitudes, and behavior related to infectious diseases in Italy
Source: PLoS One. 2019 Apr 12;14(4):e0215252. doi: 10.1371/journal.pone.0215252 (PMC6461267; doi:10.1371/journal.pone.0215252)
Supplement: S1 File — (DOCX) [file pone.0215252.s001.docx]

**QUESTIONNAIRE**

**A. SOCIO-DEMOGRAPHIC AND TRAVEL CHARACTERISTICS**

**This section is designed to gather information about your socio-demographic and travel characteristics**

**A1.** What is your gender? □ Male □ Female **A2.** How old were you on your last birthday? ________

**A3.** What is your nationality? □ Italian □ Other (please specify) **________________**

**A4.** What is your marital status? Married Single (never married) Other ___________

**A5.** How many children do you have? ______

**A6.** What is your highest education level? ____________________________________________

**A7.** What is your occupation? ___________________________________________

**A8.** What is your husband/partner highest education level? ________________________________________

**A9.** What is your husband/partner occupation? ________________________________________

**A10.** Please list the destination countries you are traveling to, in order you will visit them

__________________________________________________________________

**A11.** How many days are you planning to stay in each destination?

__________________________________________________________________

**A12.** What is the reason for your travel? □ Holiday □ Business □ Other (please specify) ___________________

**A13.** Who is accompanying you in this travel? (more answers possible) □ None □ Husband/Wife/Partner □ Children (how many____) □ Family/Relatives (how many____) □ Friends (how many____) □ Other (please specify) ______________

**A14.** Have you ever visit the following geographic areas?

|  | **Yes, how many times?** | **No** |
| --- | --- | --- |
| Africa | ___________ | □ |
| South America | ___________ | □ |
| Asia | ___________ | □ |
| Other (please specify) _______________ | ___________ | □ |

**A15.** How would you classify your current health status on a scale of 1 (bad) to 10 (excellent)?

**Bad**  1 2 3 4 5 6 7 8 9 10 **Excellent**

**B. KNOWLEDGE**

**This section is designed to explore your knowledge about the most common infectious diseases in the destination of your travel**

**B1.** What are the most common infectious diseases in the destination of your travel? (more answers possible)

|  | **Yes** | **No** | **Do not know** |
| --- | --- | --- | --- |
| Hepatitis A | □ | □ | □ |
| Diarrhea | □ | □ | □ |
| Hepatitis B | □ | □ | □ |
| Meningococcal disease | □ | □ | □ |
| Yellow fever | □ | □ | □ |
| Typhus | □ | □ | □ |
| Poliomyelitis | □ | □ | □ |
| Human Rabies | □ | □ | □ |
| Malaria | □ | □ | □ |
| Zika | □ | □ | □ |
| Chikungunya | □ | □ | □ |
| Other _________________________________________ |  |  |  |

**B2.** Which foods can cause an infection in the destination of your travel? (more answers possible)

|  | **Yes** | **No** | **Do not know** |
| --- | --- | --- | --- |
| Ice creams | □ | □ | □ |
| [Sweet/Dessert Foods](https://www.ncbi.nlm.nih.gov/pmc/articles/PMC4338308/) | □ | □ | □ |
| Undercooked meats | □ | □ | □ |
| Milk and dairy products | □ | □ | □ |
| Water and ice | □ | □ | □ |
| Fish and seafood | □ | □ | □ |
| Fruits and vegetables | □ | □ | □ |
| Eggs | □ | □ | □ |
| Other_____________________________________________ |  |  |  |

**B3.** Do you know about preventive measures recommended of the infectious diseases for your destination?

□ No □ Do not know □ Yes (please specify) __________________________________________

**C. ATTITUDES**

**This section is designed to explore your attitudes about travel-related infectious diseases**

**C1.** How do you rate the risk of getting an infectious disease during the travel?

**No risk** 1 2 3 4 5 6 7 8 9 10 **Very mucj risk**

**C2.** How would you rate the utility of prevention measures for infectious diseases before the travel?

**Useless** 1 2 3 4 5 6 7 8 9 10 **Very useful**

**D. PRACTICES**

**This section is designed to explore your practices about travel-related infectious diseases**

**D1.** Did you receive any advice regarding the measures for preventing the risk of getting an infectious disease for your destination? □ No □ Yes, from whom? ____________________________________________________

**D2.** Did you practice any preventive measure recommended for your destination?

□ No, why? (please specify) __________________________________________

□ Yes (please specify) ________________________________________________

**E. INFORMATION**

**This section is designed to know the sources and need of information about the most common infectious diseases in your destination**

**E1.** Did you receive information about the most common infectious diseases in your destination? (more answers possible)

□ No □ Yes, from whom? □ Travel Agencies □ Physicians □ Internet □ Friends □ Relatives □ Other (please specify) ________

**E2.** Did you receive information about the prevention of the infectious diseases in your destination? (more answers possible)

□ No □ Yes, from whom? □ Travel Agencies □ Physicians □ Internet □ Friends □ Relatives □ Other (please specify) ________

**E3.** Do you feel you need more information about the infectious diseases in your destination? □ No □ Yes

**The questionnaire is finished. Do you want to add something?**

**______________________________________________________________________________________________________________________________________________________________________________________________________**

**THANK YOU FOR PARTICIPATING IN THE SURVEY**
